# *Saccharomyces Cerevisiae*—An Interesting Producer of Bioactive Plant Polyphenolic Metabolites

**DOI:** 10.3390/ijms21197343

**Published:** 2020-10-05

**Authors:** Grzegorz Chrzanowski

**Affiliations:** Department of Biotechnology, Institute of Biology and Biotechnology, University of Rzeszow, 35-310 Rzeszow, Poland; gchrzanowski@ur.edu.pl; Tel.: +48-17-851-8753

**Keywords:** heterologous production, shikimic acid pathway, phenolic acids, flavonoids, anthocyanins, stilbenes

## Abstract

Secondary phenolic metabolites are defined as valuable natural products synthesized by different organisms that are not essential for growth and development. These compounds play an essential role in plant defense mechanisms and an important role in the pharmaceutical, cosmetics, food, and agricultural industries. Despite the vast chemical diversity of natural compounds, their content in plants is very low, and, as a consequence, this eliminates the possibility of the production of these interesting secondary metabolites from plants. Therefore, microorganisms are widely used as cell factories by industrial biotechnology, in the production of different non-native compounds. Among microorganisms commonly used in biotechnological applications, yeast are a prominent host for the diverse secondary metabolite biosynthetic pathways. *Saccharomyces cerevisiae* is often regarded as a better host organism for the heterologous production of phenolic compounds, particularly if the expression of different plant genes is necessary.

## 1. Introduction

Secondary metabolites are defined as valuable natural products synthesized by different organisms that are not essential for growth and development. Plants produce over 200,000 of these compounds, which mostly arise from specialized metabolite pathways.

Phenolic compounds play essential roles in interspecific competition and plant defense mechanisms against biotic and abiotic stresses [1] and radiation, and might act as regulatory molecules, pigments, or fragrances [2]. These compounds are an integral part of our daily lives, and they play important roles in the pharmaceutical, cosmetics, food, and agricultural industries. Typically, plant natural products do not show nutritional value, but a diet rich in these substances might boost the immune system [3] or decrease the level of free radicals and, thus, could prevent or suppress carcinogenesis [4].

Therefore, active compounds that are useful in medicine were intensively extracted directly from the plant material. However, these methods are often uneconomical or show destructive effects on the environment in the aftermath of harvesting the plants, particularly protected ones. Despite the vast chemical diversity of natural compounds, their content in plants can frequently be at a low level, and this eliminates the possibility of producing these interesting secondary metabolites directly from plants [5]. Interesting organic compounds are also produced via chemical synthesis. However, the structural and stereochemical complexity of distinctive plant metabolites requires sophisticated methods for the synthesis. Although the chemical industry can provide a variety of useful products, it heavily relies on crude petroleum and environmentally damaging processes [6]. However, due to the limited availability and high price of natural products, their synthetic analogues gained increasing importance in the food technology and other industries in the last decades. Following this demand, there was intensive development of the chemical industry since the 1970s [7]. In Poland alone, in the year 2018, the sale of chemicals and chemical products as well as pharmaceutical products was estimated to be at over 21 billion USD [8].

However, both the consumer’s environmental awareness and proven toxicological effects of synthetic compounds caused a higher demand for natural products [9]. Microorganisms are widely used as platform cell factories by industrial biotechnology, for the production of different non-native compounds, such as alcohols, terpenoids, alkaloids, phenylpropanoids, and polyketides [10]. Microbial production based on renewable feedstock is relatively cheap, and the intensive growth of microorganisms provides short production times. Unlike traditional, synthetic, chemistry-based routes, microbial fermentations are readily scalable from the laboratory conditions to industrial-sized bioreactors [11]. As recombinant microorganisms are typically devoid in competing pathways in relation to the heterologously expressed pathways from plants, the desired natural products are typically made in the cell, as chemically distinct substances [2].

Among the microorganisms commonly used in biotechnological applications, yeast proved to be particularly suited to host diverse secondary metabolite biosynthetic pathways [12].

In this review, I showed an extensive list of pathways leading to the biosynthesis of valuable plant phenolic secondary metabolites, starting from the synthesis of aromatic amino acids (produced via the shikimic acid pathway), through phenylpropanoid acids, flavonoids, anthocyanins, and stilbenoids. Synthetic biology significantly increased our ability of the production of natural bioactive substances in yeast. Therefore, the review is devoted to the possible application of *S. cerevisiae* for the expression of plant genes and the production of some phenolic metabolites.

## 2. Yeast Metabolism for Polyphenols Biosynthesis

### 2.1. Pyruvate and Acetyl-CoA

**Figure 1:**
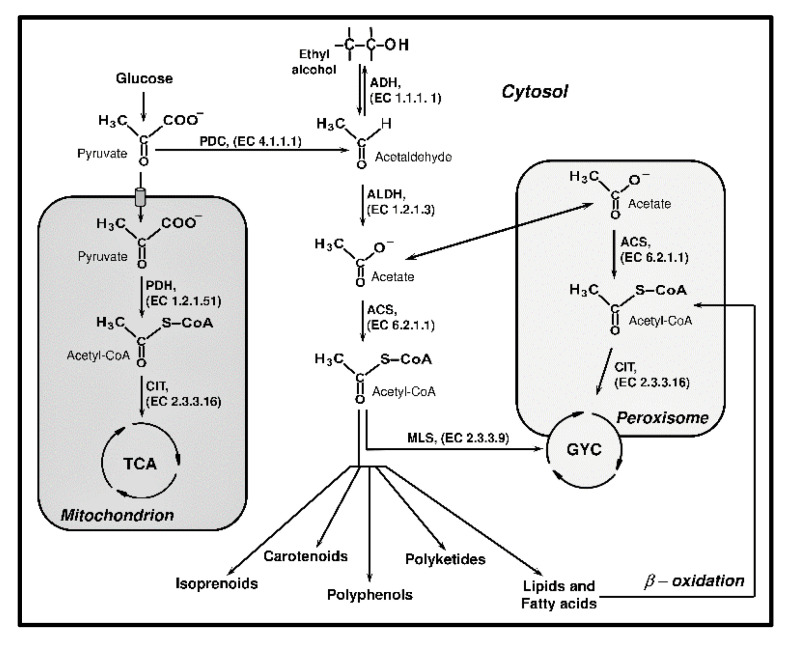
Metabolism of pyruvate and acetyl-CoA in *Saccharomyces cerevisiae*.

During growth, fungi can employ two major strategies for energy production—oxidative respiration or nonoxidative fermentation. Both respiration and fermentation employ glycolysis as the central pathway. Pyruvate is an important connection between assimilatory and dissimilatory reactions and is also the precursor in many metabolic pathways (Figure 1). In yeast, pyruvate is oxidized into carbon dioxide and water via the tricarboxylic acid cycle (TCA). Acetyl-CoA, used as the primary substrate for the TCA cycle, is generally synthesized from pyruvate during direct oxidative decarboxylation, and the reaction is catalyzed by the pyruvate dehydrogenase (PDH, EC 1.2.1.51) complex. Acetyl-CoA might also be produced from pyruvate in other indirect reactions. This pyruvate dehydrogenase bypass involves three enzymes—pyruvate decarboxylase (PDC, EC 4.1.1.1), acetaldehyde dehydrogenase (ALDH, EC 1.2.1.3), and acetyl-CoA synthetase (ACS, EC 6.2.1.1) [13].

Acetyl-CoA, as well as some intermediates synthesized in the TCA cycle, are an essential biosynthetic building block in the primary and secondary metabolite pathways. During fermentation, yeast produces different classes of compounds from the pyruvate, including isoprenoids, carotenoids, polyketides, polyphenols and lipids, and fatty acids.

However, these metabolites are synthesized by consuming cytosolic acetyl-CoA. Thus, a transporter of the mitochondria or deletion of the genes encoding the enzymes utilize acetyl-CoA in the mitochondria and peroxisome compartments. Metabolic engineering manipulations were carried out to boost the availability of acetyl-CoA in yeast [14,15].

**Figure 2:**
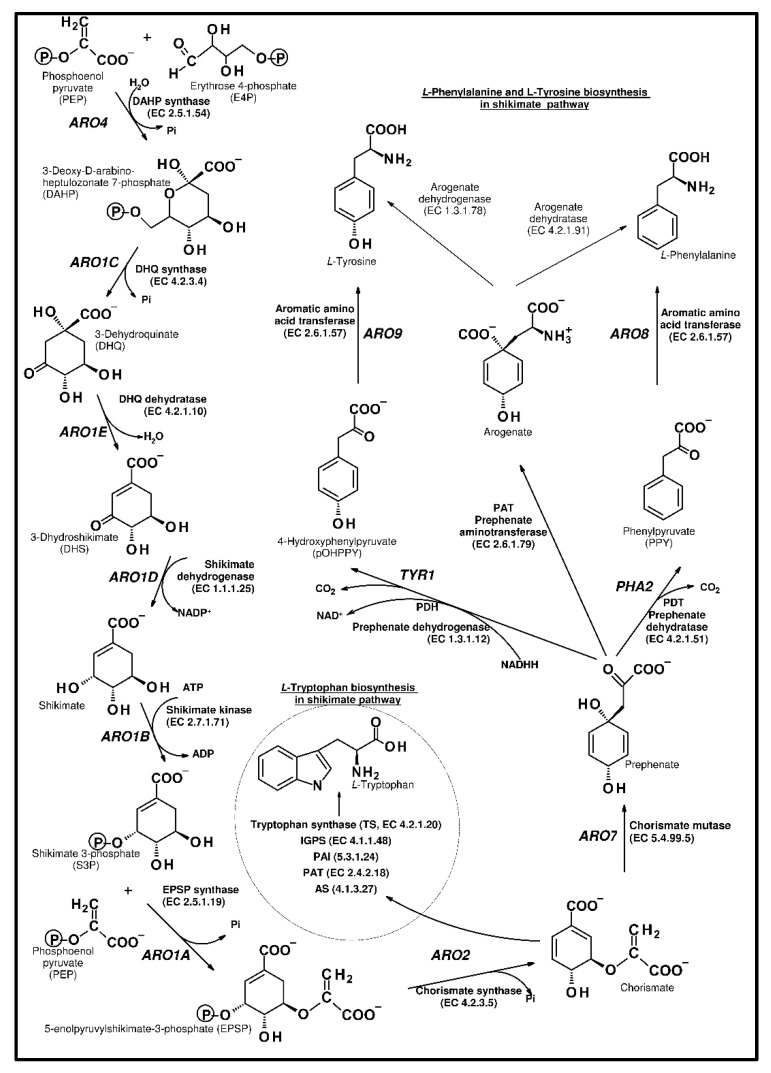
The aromatic amino acid pathway.

Microorganisms must synthesize the amino acids that are necessary for protein production. Aromatic amino acids are especially fundamental for the synthesis of these primary metabolism molecules. The pentose phosphate pathway (PPP) plays an important role in the synthesis of ribonucleotides and amino acids. *l*-phenylalanine (*l*-Phe), *l*-Tyrosine (*l*-Tyr), and *l*-tryptophan (*l*-Trp) are produced via the shikimate pathway. These aromatic acids are not only crucial components of protein biosynthesis, they are also precursors of the diverse phenolic secondary metabolites [12,16,17]. The shikimate pathway starts from erythrose 4-phosphate (E4P) obtained from glycolysis, and phosphoenolpyruvate (PEP) derived from the pentose phosphate pathway (Figure 2). The series of reactions is invariable in different organisms, including all eukaryotic and prokaryotic cells [18]. The chorismic acid is the last intermediate of all protein aromatic amino acids, and their derivatives, such as vitamin K, ubiquinone, and *p*-aminobenzoate.

Many important natural compounds are synthesized in mixed biosynthetic pathways. Therefore, the precise boundaries between the origin of individual classes of secondary metabolites are blurred. Shikimic and chorismic acids produced via the shikimic path can access the main classes of phenolic compounds, starting with simple structurally phenolic acids, such as benzoic acids, which contains only one benzoic ring (C6-C1) [19]. Phenylalanine starts the phenylpropanoid path and is the precursor of several phenylpropanoid compounds (with structure C6-C3). On the other hand, many functionalized phenylpropanoids, in particular the flavonoids, are produced in the polyketide pathway involving chain elongation by malonyl-CoA. Monoterpenoid indole alkaloids arise through condensation of the tryptamine-biogenic amine produced from tryptophan and a monoterpene-secologanin. For this biosynthesis, a strictosidine synthase (*STR*) and strictosidine β-glucosidase (*SGD*) from *Catharanthus roseus* were expressed in yeast [20]. Several other alkaloids are formed from products of the phenylpropanoid pathway, such as substituted amphetamines through the condensation of pyruvate and benzoic acid [21]. Modified amphetamines are also called phenylpropylamino alkaloids, to show their origin from phenylalanine and pyruvate. On the other hand, yeasts can produce benzylisoquinoline alkaloids (BIAs). However, the expression of methyltransferases is necessary to convert a fed substrate into reticuline. Moreover, expression of human cytochrome P450 *CYP2D6* in a reticuline-producing strain caused the synthesis of salutaridine, an intermediate in the morphine branch [22].

### 2.2. Aromatic Amino Acids and Phenolic Compounds

Aromatic amino acids are precursors of many phenolic secondary metabolites, as well as molecules like vitamins and cofactors [23]. Many of them found applications as nutraceutical and pharmaceutical ingredients. Therefore, the shikimic acid path is attractive for the discovery of biological systems and biotechnological applications in the biosynthesis of new bioactive substances [23,24].

The enzymatic steps involved in aromatic compound biosynthesis are similar in many, even genetically different, organisms, such as bacteria, fungi, and plants, but do not occur in animals. However, there are some fundamental differences connected with the regulation of the pathway and the function of enzymes [25].

In *S. cerevisiae*, the biosynthesis of the aromatic ring (Figure 2) starts from the reaction of the erythrose 4-phosphate (E4P) and phosphoenolpyruvate (PEP). DAHP synthase (EC 4.1.2.15) catalyzes this aldol condensation, and deoxy-d-arabino-heptulosonate-7-phosphate (DAHP) is produced. In yeast, two synthases (ARO3 and ARO4) were found, in the bacteria *Escherichia coli* there were three isoenzymes, AroF, AroG, and AroH, and in *Arabidopsis thaliana*, we found DAHPS1 and DAHPS2. These synthases are allosterically regulated in yeast and *l*-tyrosine regulates ARO4, whereas *l*-phenylalanine controls ARO3. In other microorganisms and plants, there is no feedback regulation; however, enzymes are activated by *l*-tryptophan [26].

In the next steps, pentafunctional ARO1 enzyme converts DAHP into 5-enolpyruvylshikimate-3-phosphate. ARO1 is a large polypeptide that represents the fusion of five different genes. This enzyme is a mosaic of five monofunctional domains and catalyzes five different reactions. ARO2 (chorismate synthase, EC 4.2.3.5) catalyzes the production of chorismate. The flavin mononucleotide is a cofactor for chorismate synthase. According to the capacity for regeneration of the cofactor, yeast chorismate synthase is a bifunctional enzyme with oxidoreductase activity [27]. At this point, the pathway divides into two branches, one connected with phenylalanine and tyrosine biosynthesis and the second toward tryptophan production [28,29,30].

The analysis and characterization of the enzymatic steps leading to the synthesis of phenylalanine in plants and bacteria showed two alternative pathways. One is similar to the yeast pathway, where phenylpyruvate is generated, following transamination to Phe. The second starts with the transamination of prephenate to arogenate, which then undergoes decarboxylation/dehydration. Thus, phenylalanine might be formed from phenylpyruvate or arogenate, whereas tyrosine synthesis proceeds from either arogenate or 4-hydroxyphenylpyruvate [31,32]. In contrast to plants and some bacterial species, in *S. cerevisiae,* only the phenylpyruvate and 4-hydroxyphenylpyruvate paths were suggested [16].

The budding yeast *S. cerevisiae* proves to be more attractive than *E. coli* bacteria, due to its robustness and tolerance to stress in the fermentative phase. It can express membrane-bound cytochrome P450 oxidases, which are key catalysts in most relevant plant-based biosynthetic pathways. Moreover, proteins produced by yeast are posttranslationally modified through the mechanisms similar to those found in plants. On the other hand, the DAHP synthase (ARO3 and ARO4) is feedback inhibited by phenylalanine and tyrosine. However, modifications of specific residues in the cavity of *ARO4* lead to a relief in feedback inhibition of the ARO4 enzyme [33,34].

The overexpression of *ARO1* and *ARO2* (chorismate synthase) in *S. cerevisiae* positively influenced the production of *p*-coumaric acid. This phenylpropenoid acid, a derivative of trans-cinnamic acid, containing a hydroxyl group at position 4, is an essential precursor for the biosynthesis of valuable natural compounds, starting from flavors and pharmaceuticals, to biocosmetics, health, and nutrition products [35]. The yeast strain overexpressing *ARO1* produced 1.69 g·dm^−3^ of *p*-coumaric acid, whereas the one overexpressing *ARO2* produced 1.41 g·dm^−3^. The simultaneous overexpression of *ARO1* and *ARO2* increased the production of *p*-coumaric acid to 1.72 g·dm^−3^. Thus, the synthesis of *p*-coumaric acid by the strains with overexpression of *ARO1* and overexpression of both the *ARO1* and *ARO2* genes, were on a similar level [25].

Later, in the branch for the synthesis of *l*-phenylalanine and *l*-tyrosine, chorismic acid is converted into prephenate (PPA) with the use of chorismate mutase (EC 5.4.99.5; ARO7). Then, phenylpyruvate is generated from prephenate, with the use of prephenate dehydratase (EC 4.2.1.51, PHA2). The generation of phenylpyruvate is processed through a decarboxylation/dehydration reaction. *S. cerevisiae* has a single *PHA2* coding sequence [36], while both the ADT1 and ADT2 *Arabidopsis* enzymes demonstrated dehydratase (PDT) activity. In *Escherichia coli*, two enzymes, prephenate dehydratase and chorismate mutase (CM EC 5.4.99.5, PDT EC 4.2.1.51), are combined in the bifunctional P-protein (PheA), and both activities are regulated by Phe-induced feedback inhibition [37]. This catalytic protein is usually encoded by the *pheA* gene [38].

In the same branch as phenylalanine, *p*-hydroxyphenylpyruvate is produced from prephenate; however, prephenate dehydrogenase activity (EC 1.3.1.12, TYR1) is used in this reaction, instead of dehydratase, during phenylpyruvate synthesis. The dehydrogenase catalyzes the reactions of oxidative carboxylation and dehydration. Similar to dehydratase in *E. coli*, a bifunctional T-protein, encoded by the *tyrA* gene [38], contains discrete separable mutase, dehydrogenase, and regulatory domains. Mutagenesis studies on the T-protein and kinetic experiments using substrate analogs suggested that the CM and PDH reactions occur at overlapping or perhaps proximal active sites [39,40].

Further, within *S. cerevisiae* cells, the 2-oxo acids can be transaminated to phenylalanine by aromatic amino acid transaminase (EC 2.6.1.57) -transferase I (ARO8) or II (ARO9) [31,41], using *L*-glutamate as the amino group donor [42]. ARO8 is mainly effective in the generation of *L*-phenylalanine and *L*-tyrosine, whereas ARO9 is involved in the catabolism of L-tryptophan. However, in strains with *ARO8* deletion, ARO9 can perform the biosynthetic function of ARO8.

Urrestarazu et al. [41] demonstrated, during in vitro research with *S. cerevisiae*, that aromatic aminotransferase I showed activity to substrates other than the aromatic amino acids. Methionine, α-aminoadipate, and leucine were also used, when phenylpyruvate was exploited as the amino group acceptor, or with their oxo-acid analogues and phenylalanine as the amino donor in the reverse reactions. Thus, this suggests that the yest aminotransferases may also take part in other metabolisms than aromatic amino acids.

**Figure 3:**
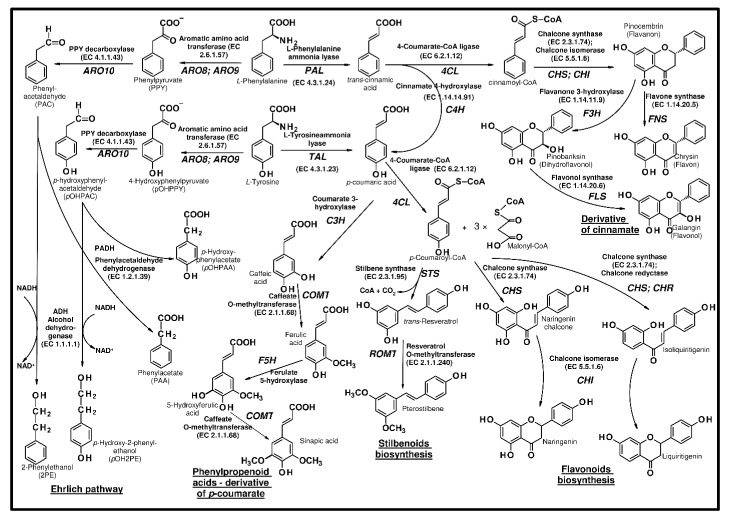
The metabolism of aromatic amino acids and the biosynthetic pathways of polyphenols.

On the other hand, many microorganisms and several yeast species produce phenylethanol (with the characteristic rose-like aroma), directly from phenylalanine or bypassing the biosynthesis of amino acids (Figure 3), via the Ehrlich pathway [43]. In *S. cerevisiae*, the decarboxylation of phenylpyruvate to phenylacetaldehyde is primarily catalyzed by thiamine pyrophosphate-dependent 2-oxo acid decarboxylase ARO10 (EC 4.1.1.43) [44]. Earlier studies [45,46] were connected with the use of *S. cerevisiae* for the production of phenylethanol using a media supplemented with phenylalanine. However, Romagnoli et al. [47] suggested that an *ARO8Δ* mutation may be useful for the de novo production of phenylethanol from glucose in ammonium-containing medium. They found that a combination of *ARO8* deletion with other mutations had a positive impact on the biosynthesis of aromatic alcohols. A combination of *ARO8* and *ARO3* deletions with the overexpression of feedback-insensitive DAHP synthase (*ARO4*) and chorismate mutase (*ARO7*) caused an increase in the concentration of the alcohols. The further deletion of the *TYR1* gene caused an increase in the phenylethanol concentration.

#### 2.2.1. Flavonoids

Among different plant secondary metabolites, flavonoids play an important role in accordance with their antioxidant, antibacterial, and anti-inflammatory activities [48,49]. According to the low concentration of flavonoids in plant sources and difficulties in the extraction of these compounds from plants, there is a great deal of interest in their production using cell factories. Many valuable flavonoids and stilbenoids were only found in a relatively small number of plant species [50]. That group constitutes a relatively diverse family of aromatic molecules that are derived from phenylalanine (or tyrosine) and malonyl-coenzyme A [51].

In plants, the biosynthesis of flavonoids (Figure 3) starts from hydroxylation of cinnamic acid to *p*-coumaric acid by trans-cinnamate 4-monooxygenase (C4H, EC 1.14.14.91), or directly from *p*-coumaric acid. These phenylpropenoid acids are produced from aromatic amino acids, via their deamination with use of ammonia lyases (PAL, EC 4.3.1.23 and TAL EC 4.3.1.24). Researchers suggested that PAL could catalyse the conversion of tyrosine into *p*-coumaric acid in the absence of C4H activity. *p*-Coumaric acid is then activated to *p*-coumaroyl-CoA by 4-coumarate-CoA ligase (4CL, EC 6.2.1.12). Chalcone synthase (CHS, EC 2.3.1.74) catalyzes the condensation of three acetate units with *p*-coumaroyl-CoA, and naringenin chalcone is formed. Chalcone synthase may catalyse the condensation of cinnamoyl-CoA or caffeoyl-CoA with malonyl-CoA, and trihydroxychalcone and pentahydroxychalcone are formed [52].

Following this reaction, chalcone isomerase (CHI, EC 5.5.1.6) performs stereospecific isomerization of tetrahydroxychalcone into (2S)-flavanone, which is the branch point precursor of many important downstream flavonoids. Different subclasses of flavonoids are generally classified as flavones, flavanols, flavonols, isoflavonoids, anthocyanins, and proanthocyanidins [19]. On the other hand, stilbene synthase (STS, EC 2.3.1.95) catalyzes the subsequent folding and cyclization of the generated tetraketide intermediate results in the production of the stilbene ring structure [52,53]. Whereas the coupled catalytic action of two polyketide enzymes, chalcone synthase and chalcone reductase generate linear di-, tri-, and tetra-ketide-CoA intermediates, yielding deoxychalcone [54,55].

**Figure 4:**
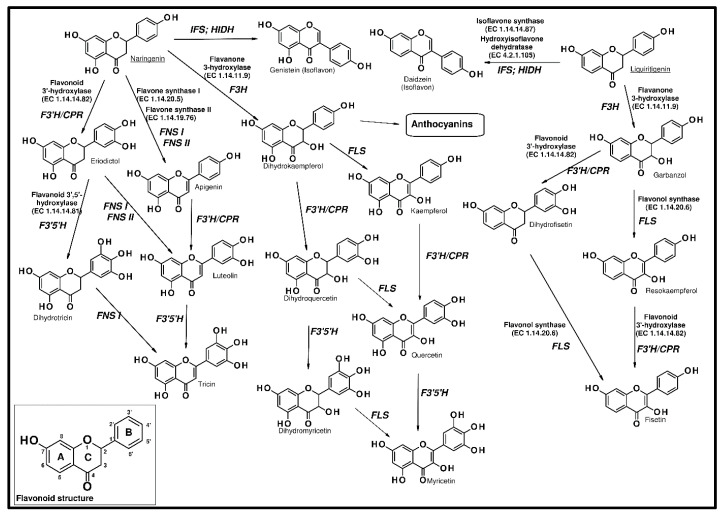
The flavonoid structure and biosynthetic pathways of major flavonoid compounds.

The subsequent transformation of naringenin (Figure 4) may produce flavanones and flavanols in reactions catalyzed by flavanone 3-hydroxylase (F3H, EC 1.14.11.9) and flavonoid 3′- hydroxylase (F3’H, EC 1.14.14.82) or flavonoid 3′ 5′ hydroxylase (F3′5′H, EC 1.14.14.81). F3H belongs to the 2-oxoglutarate-dependent dioxygenase (2ODD) family of enzymes. This enzyme catalyzes the hydroxylation of naringenin at the 3-position and produces dihydrokaempferol (DHK), belonging to dihydroflavonols (DHF). In turn, F3’H and F3’5’H, which are P450 enzymes, catalyze the hydroxylation of the B ring derived from *p*-coumaroyl-CoA, in both flavonoids and anthocyanins [56].

As previously shown, flavonoids are biosynthesized by plants. However, yeast does not naturally produce phenylpropanoid phenolics, although its metabolism provides the necessary aromatic amino acids precursors for the further phenolic biosynthesis pathway. Thus, knowledge of the microbial synthesis of these compounds is particularly attractive.

In recent years, a series of molecular biology tools have been described, and some of these techniques have already been used for the transformation of yeast for the production of valuable secondary metabolites. Generally, these yeast strains synthesize phenolic secondary metabolites by the heterologous expression of various genes from the plants and other microorganisms [57,58,59].

Ro and Douglas [60] demonstrated that the co-expression of phenylalanine ammonia-lyase (PAL), cinnamate 4-hydroxylase (C4H), and cytochrome P450 reductase (CPR) genes from poplar were caused in the production of *p*-coumaric acid by recombinant yeast. Further modification of the strains by introducing *Rhodospidrium toruloides* tyrosine ammonia-lyase (TAL) activity (that red yeast possesses a meagre PAL/TAL ratio) showed an increase in *p*-coumaric acid synthesis. They observed the conversion of tyrosine instead of phenylalanine and bypass of C4H, during the production of phenylpropenoid acid [61]. 

Yeast with overexpression of the *Flavobacterium johsoniaeu TAL* and with a double knockout of *ARO10* (phenylpyruvate decarboxylase) and *PDC5* (pyruvate decarboxylase) produced 0.55 g·dm^−3^ of *p*-coumaric acid. Whereas, yeast with a single PDC5 or ARO10 deletion produced approximately 0.3 g·dm^−3^ of that phenylpropanoid acid, in contrast to the wild-type strain, which produced 0.24 g of that coumaric acid. On the other hand, strains overexpressing *ARO4* (DAHP synthase) and *ARO7* (chorismate mutase) were able to synthesize *p*-coumaric acid. We also found that the overexpression of certain genes from *E. coli*, including dehydroquinate synthase (*AroB*), shikimate dehydrogenase (*YdiB*), and EPSP synthase (*AroA*) led to improved production of *p*-coumaric acid. However, the overexpression of shikimate kinase (*AroL*) generated the highest enhance of the synthesis of coumaric acid, reaching 1.9 g·dm^−3^ [25] (Table 1). 

Therefore, the construction of strains with the overproduction of flavonoid-intermediates is an important and essential route for further engineering of yeast capable of the biosynthesis of polyphenols and flavonoids.

Koopman et al. [59] demonstrated de novo production of naringenin (the key intermediate during flavonoid biosynthesis) from glucose, by an engineered *S. cerevisiae* strain. The combined expression of the product pathway, codon optimization, improvement of the precursor supply and reduction of byproduct formation led to concentrations of over 400 μM naringenin in aerobic, glucose-grown batch cultures. The results indicate an over four-fold increase compared with that reported in a previous Santos et al. [58] study for an engineered *E. coli* strain. This suggests that the de novo production of naringenin in *S. cerevisiae* is an important step for the production of plant-derived flavonoids from glucose.

To enable naringenin biosynthesis in *S. cerevisiae*, Koopman et al. [59] constructed two expression vectors, including the five genes required for flavonoid biosynthesis. The cytochrome P450 reductase (CPR) gene necessary for C4H activation was added to the construct, and finally, the strain IMU011 containing all these genes was built.

As the biosynthesis of aromatic amino acids is feedback inhibited by phenylalanine and tyrosine, as well as phenylethanol produced via the Ehrlich pathway, this causes a reduction of flavonoid biosynthesis, and further necessary modifications were made. In the received IMX198 strain, deletions of genes (*ARO3*, *ARO10*, *PDC5*, and *PDC6*) and the introduction of the feedback resistant DAHP synthase allele *ARO4^G226S^*, *A. thaliana* genes (*AtPAL1*, *coC4H*, *coCPR1*, *AtCHI1*, *AtCHS3*, *coCHS3*, and *At4CL3*), as well as *Rhodobacter capsulatus coTAL1* gene, were applied.

Further utilisation of naringenin by engineered yeast strains may cause the biosynthesis of genistein, kaempferol, and quercetin [62]. With the addition of naringenin as the flavonoid precursor, yeast produced kaempferol at the level of 4.6 mg·dm^−3^ after 70 h of growth, while the genistein producing strain, generated 7.7 mg·dm^−3^ of that isoflavonoid after 180 h. The lowest concentration of flavonoids, during naringenin feeding, created a quercetin producing yeast strain, and its level reached only 0.38 mg·dm^−3^ after 70 h. Jiang et al. [63] found that *S. cerevisiae* strains were able to produce naringenin and pinocembrin. However, this required an introduction of the phenylpropanoid pathway within the yeast cells. This was accomplished by the expression of phenylalanine ammonia-lyase (*PAL*) from *Rhodosporidium toruloides*, 4-coumarate-CoA ligase (*4CL*) from *A. thaliana*, and chalcone synthase (*CHS*) from *Hypericum androsaemum*.

Rodriguez et al. [64] constructed platform strains for the production of different classes of flavonoids by the modification parental yeast strains producing *p*-coumaric acid. They showed that naringenin was produced in strains with the overexpression of three genes (*4CL*, *CHS,* and *CHI*). Whereas for liquiritigenin synthesis, chalcone reductase gene (*CHR*) was additionally overexpressed. The naringenin- and liquiritigenin-producing strains were further engineered for the generation of kaempferol and resokaempferol, respectively. There were shown to overexpress the genes encoding flavanone 3-hydroxylase (*F3H*) and flavonol synthase (*FLS*). In another experiment, they constructed strains for the biosynthesis of quercetin and fisetin. However, the overexpression of cytochrome P450 flavonoid monooxygenase (*FMO*) and cytochrome P450 reductase (*CPR*) was necessary.

#### 2.2.2. Anthocyanins

**Figure 5:**
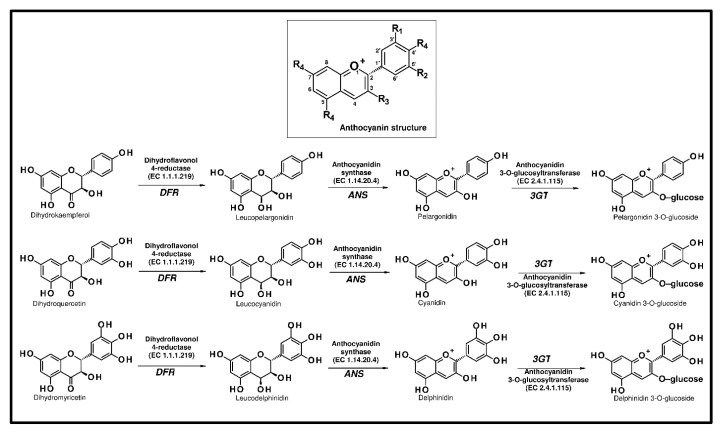
Anthocyanin structure and biosynthetic pathways (ANS, anthocyanidin synthase; DFR, dihydroflavonol 4-reductase; and 3GT, flavonoid 3-*O*-glucosyltransferase).

Anthocyanins are one of the most important plant pigments, and they are responsible for most of the red, blue, and purple colours of leaves, fruits, and flowers. Anthocyanins are considered as flavonoids due to their C6-C3-C6 chemical structure, although they have a positive charge at the oxygen atom of the C-ring of the basic flavonoid structure (Figure 5) [65].

Based on cell-line studies, animal models, and human clinical trials, researchers have suggested that anthocyanins exhibit anti-inflammatory and anti-carcinogenic activity and prevent cardiovascular diseases. They effectively diminish the level of free radicals and terminate the chain reaction that is responsible for oxidative damage [66].

Anthocyanins are produced in a specific branch of the flavonoid pathway. From naringenin, they are biosynthesized by flavanone 3-hydroxylase (F3H), dihydroflavonol 4-reductase (DFR, EC 1.1.1.219), and anthocyanidin synthase (ANS, EC 1.14.20.4). Anthocyanidin synthase has been characterized as a multifunctional protein catalyzing several reactions with different flavonoid substrate intermediates. The final reaction, converting anthocyanidins into anthocyanins, is catalyzed by an anthocyanidin 3-*O*-glucosyltransferase (3GT, EC 2.4.1.115) [71,72].

Plant cells generally produce complex mixtures of polyphenolic compounds, and these are difficult to stabilize and engineer. Levisson et al. [72], for the biosynthesis of pelargonidin 3-*O*-glucoside, used the naringenin-producing IMX106 strain previously constructed by Koopman et al. [59]. They showed the production of anthocyanins from naringenin after the subsequent insertion of the genes (*F3H*, *ANS*, and *3GT*). Further modification of the strain IMK393 showed the de novo production of kaempferol, kaempferol 3-*O*-glycoside, and pelargonidin. They introduced genes for naringenin biosynthesis and the elimination of the Ehrlich pathway. In the next step, the genes of anthocyanin synthesis were incorporated, genes encoding glucosidases were deleted, and the pathway of phloretic acid synthesis was abolished. The total sum of extracellular flavonoids at the end of the glucose consumption phase was 70.4 µM, consisting mostly of dihydrokaempferol with 59.9 µM. Whereas the concentration of flavonoids reached 202.3 µM at the end of the fermentation.

Initially, Wellman et al. [73] proposed that anthocyanidins were derived from naringenin via dihydroflavonols and leucocyanidins. These compounds might eventually be oxidized by anthocyanidin synthase (ANS). However, the investigations were conducted using *E. coli*. These experiments put into question the role of ANS, as the recombinant enzyme from *Arabidopsis* exhibited primarily flavonol synthase (FLS) activity, with negligible ANS activity. This indicated that that ANS and FLS might select dihydroflavonoid substrates for the catalyzed reaction. Recombinant ANS from *Gerbera hybrida* converted (+)-catechin into two primaries and one vestige product, whereas (−)-catechin, (−)-epicatechin, (+)-epicatechin, and (−)-gallocatechin were not accepted. The Km value for (+)-catechin was determined at 175 μM, and LC-MS and NMR analysis showed the presence of the 4,4-dimer of oxidized (+)-catechin (93%), cyanidin (7%), and traces of quercetin.

The full-length pathways for most ACN structures require the action of multiple plant CYP enzymes, and they are usually difficult to express in bacterial hosts [74]. Thus, *S. cerevisiae* is often regarded as the better host organism for the heterologous production of anthocyanins.

Eichenberger et al. [70] successfully reconstituted the full pathway of the biosynthesis of pelargonidin-3-*O*-glucoside (P3G), cyanidin-3-*O*-glucoside, and delphinidin-3-*O*-glucoside within *S. cerevisiae*. They also reported that the yeast strain used in the experiment was not specifically optimized for providing relevant precursors, and the optimization of the conditions of growth was not performed. This suggests the potential for efficient ACN production in yeast. In particular, the efficient hydroxylation of naringenin by F3΄H and F3΄5΄H demonstrates the ability of *S. cerevisiae* to functionally express plant CYPs. The concentration of eriodictyol obtained from glucose is within the same order of magnitude as the highest titers reported in *E. coli* when fed with phenylalanine [75] or caffeic acid [76]. This also suggested that the heterologous production of 5,7,3’,4’,5’-pentahydroxy-flavanone (PHF) was not reported in microorganisms.

Eichenberger et al. [70] also tested the activity of DFR, by branching off the pathway toward flavan-3-ols (F3Os), by including a leukoanthocyanidin reductase (LAR) enzyme. Certain DFRs converted dihydroflavonols into F3Os via the instable LCD intermediate, almost entirely demonstrating the high catalytic activity of both proteins. Therefore, following Yan et al. [77] and Huang et al. [78], researchers suggested that DFR might represent a rate-limiting step in the ACN pathway.

On the other hand, these results provide new information that could be used in the study of proanthocyanidins, for which F3Os are the precursors.

#### 2.2.3. Stilbenoids

Another group of compounds produced via shikimic acid and polyketide pathways are non-flavonoid polyphenolic secondary metabolites—stilbenoids. Natural stilbenes are a group of polyphenols characterized by the presence of a 1,2-diphenylethylene nucleus [79]. They are generally plant-produced substances, and, similar to flavonoids, play a defensive role against environmental stresses, such as UV radiation or fungal infection. Due to their anticancer and anti-inflammatory activities, they might be used as drugs [80]. Stilbenoids are well-known chemicals biosynthesized by bacteria. However, recombinant *S. cerevisiae* strain with introduced 4-coumaroyl-coenzyme A ligase (*4CL1*) form *Arabidopsis thaliana* and stilbene synthase (*STS*) from *Vitis vinifera* produced resveratrol, during growth on the rich medium containing *p*-coumaric acid [81].

Becker et al. [82] was the first to report the resveratrol biosynthesis ability of *S. cerevisiae*. In *p*-coumaric acid-fed strains, they introduced 4-coumarate-CoA ligase gene (*4CL*) from the hybrid poplar (*Populus trichocarpa* × *Populus deltoides*) and the gene trihydroxystilbene synthase (*STS*) from wine grapes (*Vitis vinifera*). Three years later, Zhang et al. [83] obtained resveratrol in the same yeast strain with the expression of *4CL* from *A. thaliana*, and *STS* from *V. vinifera*. Despite the apparent increase in the concentration of resveratrol in the modified strains of yeast, in *E. coli,* resveratrol was produced at a significantly higher level [84]. However, the possibility of heterologous gene expression in the yeast, as well as the modification of the media content for their growth, created the opportunity to acquire sufficient content of the synthesized components. Sydor et al. [81] found that *S. cerevisiae* expressing the *A. thaliana* 4-coumaroyl-coenzyme A ligase (*4CL1*) and the *Vitis vinifera* stilbene synthase (*STS*), with the use of a rich medium, considerably improved the resveratrol production, by up to 391 mg/L.

In another study, Li et al. [85] introduced multiple copies of the genes Ha*TAL*, At*4CL1*, and Vv*VST1* into a strain, over-expressing Sc*ARO4*^K229L^, Sc*ARO7*^G141S^, and Sc*ACC1*^S659A, S1157A^. After fed-batch cultivations, the best producer, named ST4152, finally synthesized approximately 415.65 mg per L of resveratrol in the glucose feeding phase, whereas with the feeding of ethanol, the highest titer reached 531.41 mg per L of resveratrol.

## 3. Conclusions

This review focused on the important biosynthesis pathways for aromatic amino acids and phenolic compounds, including crossing with the polyketides path. This branched pathway serves as a model system for understanding the yeast-based production of natural phenolic secondary metabolites. In particular, these compounds, according to their antioxidant, antibacterial, and anti-inflammatory activities, serve the increasing interest in developing polyphenol-rich functional foods. During recent years, the different genes involved in the biosynthesis of polyphenols were identified and characterized. However, a better understanding of their expression in yeast cell platforms is crucial to achieving the desired success.

This work described the heterologous expression of the plant genes into yeast, to enable the yeast to produce the major groups of plant-specific polyphenols. The effects of overexpression, deletion, and feedback regulation of mutually existing genes on the generation of the expected product were also shown.

The synthesis of natural compounds from a cheap carbon source through microbial fermentation is attractive due to the short process time, feedstock uniformity, and high purity of the product. However, after establishing the appropriate pathway in the yeast and demonstrating the production of the desired metabolite, further optimization to obtain a desirable product yield is necessary.

The author of this review hopes that this paper highlights the importance and advantages of phenylpropanoids, flavonoids, anthocyanins, and stilbenes production by yeast, in order to promote further research in this field.

## Figures and Tables

**Table 1:** Gene modification and the formation of polyphenolic compounds through recombinant *Saccharomyces cerevisiae* strains.

Metabolite	Yeast Strain	Productivity	Genes Modification ^1^	References
*p*-Coumaric acid	ST4067	1.71 g·dm^−3^	*ARO10*Δ, *PDC5*Δ, Fj*TAL*,Sc*ARO4*^fbr^, Sc*ARO7*^fbr^, Sc*ARO1*, Sc*ARO2*	Rodriguez et al. (2015) [25]
Liquiritigenin	ST5069	5.31 mg·dm^−3^	*ARO10*Δ, *PDC5*Δ, Fj*TAL*,Sc*ARO4*^fbr^, Sc*ARO7*^fbr^, Pc*4CL*, Ph*CHS*, Ms*CHI*, Amo*CHR*	Rodriguez et al. (2017) [64]
Kaempferol	ST5070	26.57 mg·dm^−3^	*ARO10*Δ, *PDC5*Δ, Fj*TAL*,Sc*ARO4*^fbr^, Sc*ARO7*^fbr^, Pc*4CL*, Ph*CHS*, Ms*CHI*, Am*F3H*, At*FLS*	Rodriguez et al. (2017) [64]
Quercetin	ST5074	20.38 mg·dm^−3^	*ARO10*Δ, *PDC5*Δ, Fj*TAL*,Sc*ARO4*^fbr^, Sc*ARO7*^fbr^, Pc*4CL*, Ph*CHS*, Ms*CHI*, Am*F3H*, At*FLS*, Cr*CPR*, Ph*F3’H*	Rodriguez et al. (2017) [64]
Breviscapine (scutellarin and apigenin-7-O-glucuronide)	ΔMC-FU-FC-AAA	108 mg·dm^−3^ and 185 mg·dm^−3^	Eb*4CL*, Eb*CHS*, Eb*CHI*, Eb*FNSII*, Eb*PAL*, Eb*C4H*, Eb*F6H*, Eb*CPR*, Eb*F7GAT*, Eb*UDPGDH*, *mls1*Δ, *cit2*Δ, Se*ACS*, *ALDH6*, *ADH2*	Liu et al. (2018) [67]
Resveratrol	ST4990	272.6 mg·dm^−3^	At*PAL2*, At*C4H*, At*4CL2*,Vv*VST1*, *ACC*, *ARO7* ^fbr^, *ARO4* ^fbr^, *ARO10*Δ, Se*ACS*, At*ATR2*, Sc*CYB5*	Li et al. (2016) [68]
Pterostilbene	ST4994	5.5 mg·dm^−3^ (mineral medium) 34.9 mg·dm^−3^ (FIT medium)	At*PAL2*, At*C4H*, At*4CL2*,Vv*VST1*, *ACC*, *ARO7* ^fbr^, *ARO4* ^fbr^, *ARO10*Δ, Se*ACS*, At*ATR2*, Sc*CYB5*, Vv*ROMT*	Li et al. (2016) [68]
Pinocembrin	Yeast, harboring plasmid Ycc4c-181	16.3 mg·dm^−3^ (from cinnamic acid)	At*C4H*, Pc*4CL2*, Ph*CHS*, Ph*CHI*	Yan et al. (2005) [69]
Cyanidin 3-Oglucoside	CANS3	2.0 mg·dm^−3^	Ha*CHS*, Ms*CHI*, At*4CL2*, At*PAL2*, Am*C4H*, Sc*CPR1*, Ph*F3’H*, At*CPR1*, Md*ANS*, Fa*A3GT2*, Md*F3H*, Pt*DFR*	Eichenberger et al. (2018) [70]
Delphinidin 3-Oglucoside	DANS6	2.1 mg·dm^−3^	Ha*CHS*, Ms*CHI*, At*4CL2*, At*PAL2*, Am*C4H*, Sc*CPR1*, Sl*F3’5’H*, At*CPR1*, Pco*ANS*, Fa*A3GT2*, Md*F3H*	Eichenberger et al. (2018) [70]

^1^ 4CL, 4-coumarate-CoA ligase; 4CL2, 4-coumarate-CoA ligase 2; A3GT2, Anthocyanidin 3-O-glucosyltransferase; ACC, Acetyl-CoA carboxylase; ACS, Acetyl-CoA synthetase; ADH2, Alcohol dehydrogenase; ALDH6, acetaldehyde dehydrogenase; ANS, Anthocyanidin synthase; ARO1, pentafunctional enzyme converting DAHP into 5-enolpyruvylshikimate-3-phosphate; ARO2, Chorismate synthase; ARO4, Deoxy-D-arabino-heptulosonate-7-phosphate synthase (DAHP synthase); ARO7, Chorismate mutase; ARO10, 2-oxo acid decarboxylase; ATR2, Cytochrome P450 reductase; C4H, Cinnamate-4-hydroxylase; CHI, Chalcone isomerase; CHR, Chalcone reductase; CHS, Chalcone synthase; CIT2, Citrate synthase; CPR, Cytochrome P450 reductase; CPR1, Cytochrome P450 reductase; CYB5, Cytochrome-b5 reductase; DFR, Dihydroflavonol-4-reductase; F3’5’H, Flavonoid 3’ 5’ hydroxylase; F3’H, Flavonoid 3’-hydroxylase; F3H, Flavanone 3-hydroxylase; F6H, Flavone-6-hydroxylase; F7GAT, Flavonoid-7-O-glucuronosyltransferase; FLS, Flavonol synthase; FNSII, Flavone synthase II; MLS1, Malate synthase; PAL, Phenylalanine ammonia lyase; PAL2, Phenylalanine ammonia lyase 2; PDC5, Pyruvate decarboxylase; ROMT, Resveratrol O-methyltransferases; TAL, *l*-tyrosine ammonia lyase; UDPGDH, UDP-glucose dehydrogenase; VST1, stilbene synthase; Am, *Ammi majus*; Amo, *Astragalus mongholicus*; At, *Arabidopsis thaliana*; Cr, *Catharanthus roseus*; Eb, *Erigeron breviscapus*; Fa, *Fragaria x ananassa*; Fj, *Flavobacterium johnsoniae*; Ha, *Hypericum androsaemum*; Md, *Malus x domestica*; Ms, *Medicago sativa*; Pc, *Petroselinum crispum*; Pco, *Pyrus communis*; Ph, *Petunia x hybrida*; Pt, *Populus trichocarpa*; Sc, *Saccharomyces cerevisiae*; Se, *Salmonella enterica*; Sl, *Solanum lycopersicum*; Vv, *Vitis vinifera*; and ^fbr^, Feedback-inhibition resistant.

## Abbreviations

4CL	4-coumarate-CoA ligase
4CL2	4-coumarate-CoA ligase 2
A3GT2	Anthocyanidin 3-O-glucosyltransferase
ACC	Acetyl-CoA carboxylase
ACS	Acetyl-CoA synthetase
ADH2	Alcohol dehydrogenase
ALDH6	Acetaldehyde dehydrogenase
ANS	Anthocyanidin synthase
ARO1	Pentafunctional enzyme converting DAHP into 5-enolpyruvylshikimate-3-phosphate
ARO2	Chorismate synthase
ARO4	Deoxy-d-arabino-heptulosonate-7-phosphate synthase (DAHP synthase)
ARO7	Chorismate mutase
ARO8	Aromatic amino acid transferase
ARO9	Aromatic amino acid transferase
ARO10	2-oxo acid decarboxylase
ATR2	Cytochrome P450 reductase
C4H	Cinnamate-4-hydroxylase
CHI	Chalcone isomerase
CHR	Chalcone reductase
CHS	Chalcone synthase
CIT2	Citrate synthase
CPR	Cytochrome P450 reductase
CPR1	Cytochrome P450 reductase
CYB5	Cytochrome-b5 reductase
DFR	Dihydroflavonol-4-reductase
F3’5’H	Flavonoid 3’ 5’ hydroxylase
F3’H	Flavonoid 3’-hydroxylase
F3H	Flavanone 3-hydroxylase
F6H	Flavone-6-hydroxylase
F7GAT	Flavonoid-7-O-glucuronosyltransferase
FLS	Flavonol synthase
FNSI	Flavone synthase I
FNSII	Flavone synthase II
MLS1	Malate synthase
PAL	Phenylalanine ammonia lyase
PAL2	Phenylalanine ammonia lyase 2
PDC5	Pyruvate decarboxylase
ROMT	Resveratrol O-methyltransferases
TAL	*l*-tyrosine ammonia lyase
UDPGDH	UDP-glucose dehydrogenase
VST1	stilbene synthase
Am	*Ammi majus*
Amo	*Astragalus mongholicus*
At	*Arabidopsis thaliana*
Cr	*Catharanthus roseus*
Eb	*Erigeron breviscapus*
Fa	*Fragaria x ananassa*
Fj	*Flavobacterium johnsoniae*
Ha	*Hypericum androsaemum*
Md	*Malus x domestica*
Ms	*Medicago sativa*
Pc	*Petroselinum crispum*
Pco	*Pyrus communis*
Ph	*Petunia x hybrida*
Pt	*Populus trichocarpa*
Sc	*Saccharomyces cerevisiae*
Se	*Salmonella enterica*
Sl	*Solanum lycopersicum*
Vv	*Vitis vinifera*
fbr	Feedback-inhibition resistant
co	Codon optimized

## References

[1] Czerniewicz P., Sytykiewicz H., Durak R., Borowiak-Sobkowiak B., Chrzanowski G. (2017). Role of phenolic compounds during antioxidative responses of winter triticale to aphid and beetle attack. Plant Physiol. Biochem..

[2] Marienhagen J., Bott M. (2013). Metabolic engineering of microorganisms for the synthesis of plant natural products. J. Biotechnol..

[3] Licciardi P.V., Underwood J.R. (2011). Plant-derived medicines: A novel class of immunological adjuvants. Int. Immunopharmacol..

[4] Tan A.C., Konczak I., Sze D.M.-Y., Ramzan I. (2011). Molecular Pathways for Cancer Chemoprevention by Dietary Phytochemicals. Nutr. Cancer.

[5] Atanasov A.G., Waltenberger B., Pferschy-Wenzig E.-M., Linder T., Wawrosch C., Uhrin P., Temml V., Wang L., Schwaiger S., Heiss E.H. (2015). Discovery and resupply of pharmacologically active plant-derived natural products: A review. Biotechnol. Adv..

[6] Dhamankar H., Prather K.L. (2011). Microbial chemical factories: Recent advances in pathway engineering for synthesis of value added chemicals. Curr. Opin. Struct. Biol..

[7] Chen Y., Nielsen J. (2013). Advances in metabolic pathway and strain engineering paving the way for sustainable production of chemical building blocks. Curr. Opin. Biotechnol..

[8] GUS Production of Industrial Products in 2018. https://stat.gov.pl/en/topics/industry-construction-fixed-assets/industry/production-of-industrial-products-in-2018,9,2.html.

[9] Sowbhagya H.B., Chitra V.N. (2010). Enzyme-Assisted Extraction of Flavorings and Colorants from Plant Materials. Crit. Rev. Food Sci. Nutr..

[10] Nielsen J. (2014). Synthetic Biology for Engineering Acetyl Coenzyme A Metabolism in Yeast. mBio.

[11] Walker R.S.K., Pretorius I.S. (2018). Applications of Yeast Synthetic Biology Geared towards the Production of Biopharmaceuticals. Genes.

[12] Pyne M.E., Narcross L., Martin V.J.J. (2019). Engineering Plant Secondary Metabolism in Microbial Systems. Plant Physiol..

[13] Pronk J.T., Steensma H.Y., Van Dijken J.P. (1996). Pyruvate Metabolism in Saccharomyces cerevisiae. Yeast.

[14] Strijbis K., Distel B. (2010). Intracellular Acetyl Unit Transport in Fungal Carbon Metabolism. Eukaryot. Cell.

[15] Chen Y., Daviet L., Schalk M., Siewers V., Nielsen J. (2013). Establishing a platform cell factory through engineering of yeast acetyl-CoA metabolism. Metabol. Eng..

[16] Braus G.H. (1991). Aromatic amino acid biosynthesis in the yeast Saccharomyces cerevisiae: A model system for the regulation of a eukaryotic biosynthetic pathway. Microbiol. Rev..

[17] Kobayashi Y., Sahara T., Ohgiya S., Kamagata Y., Fujimori K.E. (2018). Systematic optimization of gene expression of pentose phosphate pathway enhances ethanol production from a glucose/xylose mixed medium in a recombinant Saccharomyces cerevisiae. AMB Exp..

[18] Carlquist M., Gibson B., Yuceer Y.K., Paraskevopoulou A., Sandell M., Angelov A.I., Gotcheva V., Angelov A.D., Etschmann M., de Billerbeck G.M. (2015). Process engineering for bioflavour production with metabolically active yeasts—A mini-review. Yeast.

[19] Ferrer J.-L., Austin M.B., Stewart C., Noel J.P. (2008). Structure and function of enzymes involved in the biosynthesis of phenylpropanoids. Plant Physiol. Biochem..

[20] Geerlings A., Redondo F., Contin A., Memelink J., van der Heijden R., Verpoorte R. (2001). Biotransformation of tryptamine and secologanin into plant terpenoid indole alkaloids by transgenic yeast. Appl. Microbiol. Biotechnol..

[21] Hagel J.M., Krizevski R., Marsolais F., Lewinsohn E., Facchini P.J. (2012). Biosynthesis of amphetamine analogs in plants. Trends Plant Sci..

[22] Hawkins K.M., Smolke C.D. (2008). Production of benzylisoquinoline alkaloids in Saccharomyces cerevisiae. Nat. Chem. Biol..

[23] Lamichhane G., Freundlich J.S., Ekins S., Wickramaratne N., Nolan S.T., Bishai W.R. (2011). Essential Metabolites of Mycobacterium tuberculosis and Their Mimics. mBio.

[24] Scotti L., Bezerra Mendonça Junior F.J., Magalhaes Moreira D.R., da Silva M.S., Pitta I.R., Scotti M.T. (2012). SAR, QSAR and docking of anticancer flavonoids and variants: A review. Curr. Top. Med. Chem..

[25] Rodriguez A., Kildegaard K.R., Li M., Borodina I., Nielsen J. (2015). Establishment of a yeast platform strain for production of p-coumaric acid through metabolic engineering of aromatic amino acid biosynthesis. Metabol. Eng..

[26] Maeda H., Dudareva N. (2012). The Shikimate Pathway and Aromatic Amino Acid Biosynthesis in Plants. Ann. Rev. Plant Biol..

[27] Quevillon-Cheruel S., Leulliot N., Meyer P., Graille M., Bremang M., Blondeau K., Sorel I., Poupon A., Janin J., Tilbeurgh H. (2004). van Crystal Structure of the Bifunctional Chorismate Synthase from Saccharomyces cerevisiae. J. Biol. Chem..

[28] Duncan K., Edwards R.M., Coggins J.R. (1988). The Saccharomyces cerevisiae ARO1 gene An example of the co-ordinate regulation of five enzymes on a single biosynthetic pathway. FEBS Lett..

[29] Jones D.G.L., Reusser U., Braus G.H. (1991). Molecular cloning, characterization and analysis of the regulation of the AR02 gene, encoding chorismate synthase, of Saccharomyces cerevisiae. Mol. Microbiol..

[30] Helmstaedt K., Strittmatter A., Lipscomb W.N., Braus G.H. (2005). Evolution of 3-deoxy-d-arabino-heptulosonate-7-phosphate synthase-encoding genes in the yeast Saccharomyces cerevisiae. PNAS.

[31] Bross C.D., Corea O.R.A., Kaldis A., Menassa R., Bernards M.A., Kohalmi S.E. (2011). Complementation of the pha2 yeast mutant suggests functional differences for arogenate dehydratases from Arabidopsis thaliana. Plant Physiol. Biochem..

[32] Yoo H., Widhalm J.R., Qian Y., Maeda H., Cooper B.R., Jannasch A.S., Gonda I., Lewinsohn E., Rhodes D., Dudareva N. (2013). An alternative pathway contributes to phenylalanine biosynthesis in plants via a cytosolic tyrosine:phenylpyruvate aminotransferase. Nat. Commun..

[33] Hartmann M., Schneider T.R., Pfeil A., Heinrich G., Lipscomb W.N., Braus G.H. (2003). Evolution of feedback-inhibited β/α barrel isoenzymes by gene duplication and a single mutation. PNAS.

[34] Gottardi M., Reifenrath M., Boles E., Tripp J. (2017). Pathway engineering for the production of heterologous aromatic chemicals and their derivatives in Saccharomyces cerevisiae: Bioconversion from glucose. FEMS Yeast Res..

[35] Liu Q., Yu T., Li X., Chen Y., Campbell K., Nielsen J., Chen Y. (2019). Rewiring carbon metabolism in yeast for high level production of aromatic chemicals. Nat. Commun..

[36] Maftahi M., Nicaud J.-M., Levesque H., Gaillardin C. (1995). Sequencing analysis of a 24·7 kb fragment of yeast chromosome XIV identifies six known genes, a new member of the hexose transporter family and ten new open reading frames. Yeast.

[37] Zhang S., Wilson D.B., Ganem B. (2000). Probing the Catalytic Mechanism of Prephenate Dehydratase by Site-Directed Mutagenesis of the *Escherichia coli* P-Protein Dehydratase Domain ^†^. Biochemistry.

[38] Parthasarathy A., Cross P.J., Dobson R.C.J., Adams L.E., Savka M.A., Hudson A.O. (2018). A Three-Ring Circus: Metabolism of the Three Proteogenic Aromatic Amino Acids and Their Role in the Health of Plants and Animals. Front. Mol. Biosci..

[39] Vincent S., Chen S., Wilson D.B., Ganem B. (2002). Probing the overlap of chorismate mutase and prephenate dehydrogenase sites in the escherichia coli T-protein: A dehydrogenase-selective inhibitor. Bioorgan. Med. Chem. Lett..

[40] Hassing E.-J., de Groot P.A., Marquenie V.R., Pronk J.T., Daran J.-M.G. (2019). Connecting central carbon and aromatic amino acid metabolisms to improve de novo 2-phenylethanol production in Saccharomyces cerevisiae. Metabol. Eng..

[41] Urrestarazu A., Vissers S., Iraqui I., Grenson M. (1998). Phenylalanine- and tyrosine-auxotrophic mutants of Saccharomyces cerevisiae impaired in transamination. Mol. Gen. Genet..

[42] Enzyme Database—BRENDA. https://www.brenda-enzymes.org/.

[43] Hazelwood L.A., Daran J.-M., van Maris A.J.A., Pronk J.T., Dickinson J.R. (2008). The Ehrlich pathway for fusel alcohol production: A century of research on Saccharomyces cerevisiae metabolism. Appl. Environ. Microbiol..

[44] Vuralhan Z., Luttik M.A.H., Tai S.L., Boer V.M., Morais M.A., Schipper D., Almering M.J.H., Kötter P., Dickinson J.R., Daran J.-M. (2005). Physiological characterization of the ARO10-dependent, broad-substrate-specificity 2-oxo acid decarboxylase activity of Saccharomyces cerevisiae. Appl. Environ. Microbiol..

[45] Cui Z., Yang X., Shen Q., Wang K., Zhu T. (2011). Optimisation of biotransformation conditions for production of 2-phenylethanol by a Saccharomyces cerevisiae CWY132 mutant. Nat. Prod. Res..

[46] Kim B., Cho B.-R., Hahn J.-S. (2014). Metabolic engineering of Saccharomyces cerevisiae for the production of 2-phenylethanol via Ehrlich pathway. Biotechnol. Bioeng..

[47] Romagnoli G., Knijnenburg T.A., Liti G., Louis E.J., Pronk J.T., Daran J.-M. (2015). Deletion of the Saccharomyces cerevisiae ARO8 gene, encoding an aromatic amino acid transaminase, enhances phenylethanol production from glucose. Yeast.

[48] Xu Y.-B., Chen G.-L., Guo M.-Q. (2019). Antioxidant and Anti-Inflammatory Activities of the Crude Extracts of Moringa oleifera from Kenya and Their Correlations with Flavonoids. Antioxidants (Basel).

[49] Adamczak A., Ożarowski M., Karpiński T.M. (2020). Antibacterial Activity of Some Flavonoids and Organic Acids Widely Distributed in Plants. J. Clin. Med..

[50] Ververidis F., Trantas E., Douglas C., Vollmer G., Kretzschmar G., Panopoulos N. (2007). Biotechnology of flavonoids and other phenylpropanoid-derived natural products. Part I: Chemical diversity, impacts on plant biology and human health. Biotechnol. J..

[51] Winkel-Shirley B. (2001). Flavonoid Biosynthesis. A Colorful Model for Genetics, Biochemistry, Cell Biology, and Biotechnology. Plant Physiol..

[52] Petrussa E., Braidot E., Zancani M., Peresson C., Bertolini A., Patui S., Vianello A. (2013). Plant Flavonoids—Biosynthesis, Transport and Involvement in Stress Responses. Int. J. Mol. Sci..

[53] Watts K.T., Lee P.C., Schmidt-Dannert C. (2006). Biosynthesis of plant-specific stilbene polyketides in metabolically engineered Escherichia coli. BMC Biotechnol..

[54] Bomati E.K., Austin M.B., Bowman M.E., Dixon R.A., Noel J.P. (2005). Structural Elucidation of Chalcone Reductase and Implications for Deoxychalcone Biosynthesis. J. Biol. Chem..

[55] Mameda R., Waki T., Kawai Y., Takahashi S., Nakayama T. (2018). Involvement of chalcone reductase in the soybean isoflavone metabolon: Identification of GmCHR5, which interacts with 2-hydroxyisoflavanone synthase. Plant J..

[56] Tanaka Y., Sasaki N., Ohmiya A. (2008). Biosynthesis of plant pigments: Anthocyanins, betalains and carotenoids. Plant J..

[57] Leonard E., Lim K.-H., Saw P.-N., Koffas M.A.G. (2007). Engineering Central Metabolic Pathways for High-Level Flavonoid Production in Escherichia coli. Appl Environ. Microbiol..

[58] Santos C.N.S., Koffas M., Stephanopoulos G. (2011). Optimization of a heterologous pathway for the production of flavonoids from glucose. Metabol. Eng..

[59] Koopman F., Beekwilder J., Crimi B., van Houwelingen A., Hall R.D., Bosch D., van Maris A.J., Pronk J.T., Daran J.-M. (2012). De novo production of the flavonoid naringenin in engineered Saccharomyces cerevisiae. Microb. Cell Factories.

[60] Ro D.-K., Douglas C.J. (2004). Reconstitution of the Entry Point of Plant Phenylpropanoid Metabolism in Yeast (*Saccharomyces cerevisiae*) implications for control of metabolic flux into the phenylpropanoid pathway. J. Biol. Chem..

[61] Vannelli T., Wei Qi W., Sweigard J., Gatenby A.A., Sariaslani F.S. (2007). Production of p-hydroxycinnamic acid from glucose in Saccharomyces cerevisiae and Escherichia coli by expression of heterologous genes from plants and fungi. Metabol. Eng..

[62] Trantas E., Panopoulos N., Ververidis F. (2009). Metabolic engineering of the complete pathway leading to heterologous biosynthesis of various flavonoids and stilbenoids in Saccharomyces cerevisiae. Metabol. Eng..

[63] Jiang H., Wood K.V., Morgan J.A. (2005). Metabolic Engineering of the Phenylpropanoid Pathway in Saccharomyces cerevisiae. Appl. Environ. Microbiol..

[64] Rodriguez A., Strucko T., Stahlhut S.G., Kristensen M., Svenssen D.K., Forster J., Nielsen J., Borodina I. (2017). Metabolic engineering of yeast for fermentative production of flavonoids. Bioresour. Technol..

[65] Kong J.-M., Chia L.-S., Goh N.-K., Chia T.-F., Brouillard R. (2003). Analysis and biological activities of anthocyanins. Phytochemistry.

[66] He J., Giusti M.M. (2010). Anthocyanins: Natural Colorants with Health-Promoting Properties. Annu. Rev. Food Sci. Technol..

[67] Liu X., Cheng J., Zhang G., Ding W., Duan L., Yang J., Kui L., Cheng X., Ruan J., Fan W. (2018). Engineering yeast for the production of breviscapine by genomic analysis and synthetic biology approaches. Nat. Commun..

[68] Li M., Schneider K., Kristensen M., Borodina I., Nielsen J. (2016). Engineering yeast for high-level production of stilbenoid antioxidants. Sci. Rep..

[69] Yan Y., Kohli A., Koffas M.A.G. (2005). Biosynthesis of Natural Flavanones in Saccharomyces cerevisiae. Appl. Environ. Microbiol..

[70] Eichenberger M., Hansson A., Fischer D., Dürr L., Naesby M. (2018). De novo biosynthesis of anthocyanins in Saccharomyces cerevisiae. FEMS Yeast Res..

[71] Petroni K., Tonelli C. (2011). Recent advances on the regulation of anthocyanin synthesis in reproductive organs. Plant Sci..

[72] Levisson M., Patinios C., Hein S., de Groot P.A., Daran J.-M., Hall R.D., Martens S., Beekwilder J. (2018). Engineering de novo anthocyanin production in Saccharomyces cerevisiae. Microbial. Cell Factories.

[73] Wellmann F., Griesser M., Schwab W., Martens S., Eisenreich W., Matern U., Lukačin R. (2006). Anthocyanidin synthase from Gerbera hybrida catalyzes the conversion of (+)-catechin to cyanidin and a novel procyanidin. FEBS Lett..

[74] Kim Y.H., Kwon T., Yang H.J., Kim W., Youn H., Lee J.Y., Youn B. (2011). Gene engineering, purification, crystallization and preliminary X-ray diffraction of cytochrome P450 *p*-coumarate-3-hydroxylase (C3H), the *Arabidopsis* membrane protein. Protein Expression and Purification.

[75] Zhu S., Wu J., Du G., Zhou J., Chen J. (2014). Efficient Synthesis of Eriodictyol from l-Tyrosine in Escherichia coli. Appl. Environ. Microbiol..

[76] Fowler Z.L., Gikandi W.W., Koffas M.A.G. (2009). Increased Malonyl Coenzyme A Biosynthesis by Tuning the Escherichia coli Metabolic Network and Its Application to Flavanone Production. Appl. Environ. Microbiol..

[77] Yan Y., Li Z., Koffas M.A.G. (2008). High-yield anthocyanin biosynthesis in engineered *Escherichia coli*. Biotechnol. Bioeng..

[78] Huang Y., Gou J., Jia Z., Yang L., Sun Y., Xiao X., Song F., Luo K. (2012). Molecular Cloning and Characterization of Two Genes Encoding Dihydroflavonol-4-Reductase from Populus trichocarpa. PLoS ONE.

[79] Rivière C., Pawlus A.D., Mérillon J.-M. (2012). Natural stilbenoids: Distribution in the plant kingdom and chemotaxonomic interest in Vitaceae. Nat. Prod. Rep..

[80] Sirerol J.A., Rodríguez M.L., Mena S., Asensi M.A., Estrela J.M., Ortega A.L. (2016). Role of Natural Stilbenes in the Prevention of Cancer. Oxid. Med. Cell. Longev..

[81] Sydor T., Schaffer S., Boles E. (2010). Considerable Increase in Resveratrol Production by Recombinant Industrial Yeast Strains with Use of Rich Medium. Appl. Environ. Microbiol..

[82] Becker J.V.W., Armstrong G.O., Van Der Merwe M.J., Lambrechts M.G., Vivier M.A., Pretorius I.S. (2003). Metabolic engineering of Saccharomyces cerevisiae for the synthesis of the wine-related antioxidant resveratrol. FEMS Yeast Res..

[83] Zhang Y., Li S.-Z., Li J., Pan X., Cahoon R.E., Jaworski J.G., Wang X., Jez J.M., Chen F., Yu O. (2006). Using Unnatural Protein Fusions to Engineer Resveratrol Biosynthesis in Yeast and Mammalian Cells. J. Am. Chem. Soc..

[84] Beekwilder J., Wolswinkel R., Jonker H., Hall R., de Vos C.H.R., Bovy A. (2006). Production of Resveratrol in Recombinant Microorganisms. Appl. Environ. Microbiol..

[85] Li M., Kildegaard K.R., Chen Y., Rodriguez A., Borodina I., Nielsen J. (2015). De novo production of resveratrol from glucose or ethanol by engineered Saccharomyces cerevisiae. Metabol. Eng..

